# HSCARG Inhibits NADPH Oxidase Activity through Regulation of the Expression of p47phox

**DOI:** 10.1371/journal.pone.0059301

**Published:** 2013-03-19

**Authors:** Weichun Xiao, Yanyan Peng, Yong Liu, Zhi Li, Senlin Li, Xiaofeng Zheng

**Affiliations:** 1 State Key Lab of Protein and Plant Gene Research, Beijing, China; 2 Department of Biochemistry and Molecular Biology, School of Life Sciences, Peking University, Beijing, China; 3 Department of Medicine, University of Texas Health Science Center and South Texas Veterans Health Care System, Audie L. Murphy Division, San Antonio, Texas, United States of America; Baylor College of Medicine, United States of America

## Abstract

Nicotinamide adenine dinucleotide phosphate (NADPH) oxidase catalyzes the transfer of electrons from NADPH to O_2_, which is the main source of reactive oxygen species (ROS) in nonphagocytic cells. Excess ROS are toxic; therefore, keeping ROS in homeostasis in cells can protect cells from oxidative damage. It is meaningful to further understand the molecular mechanism by which ROS homeostasis is mediated. Human protein HSCARG is a newly identified oxidative sensor and a negative regulator of NF-κB. Here, we find that HSCARG represses the cellular ROS generation through inhibiting mRNA and protein expression of p47phox, a subunit of NADPH oxidase. In contrast, shRNA-mediated HSCARG knockdown increases endogenous p47phox expression level. And HSCARG has no obvious effect on ROS production in p47phox-depleted cells. Furthermore, HSCARG regulates p47phox through inhibition of NF-κB activity. Our findings identify HSCARG as a novel regulator in regulation of the activity of NADPH oxidase and ROS homeostasis.

## Introduction

Reactive oxygen species (ROS) include oxygen radicals such as superoxide (O_2_^−^), hydroxyl (OH), peroxyl (RO_2_), alkoxyl (RO), and certain nonradicals such as singlet oxygen (O_2_), and hydrogen peroxide (H_2_O_2_) [1]. They are produced via various processes including mitochondrial electron transport chain, nitric oxide synthase, xanthine oxidase, as well as nicotinamide adenine dinucleotide phosphate (NADPH) oxidase [1], [2]. NADPH oxidase, also termed as NADPH oxidase (NOX) family, contains distinct Nox subunits (Nox1–5, DUOX1, DUOX2) and is the main source of ROS in nonphagocytic cells [1], [3]. NADPH oxidase components include membrane-bound heterodimer (NOX and p22phox) and four cytosolic proteins including p47phox, p67phox, p40phox and Rac1/2 [4]. The four cytosolic subunits are activated and translocated to cell membrane where they interact with the heterodimer (NOX and p22phox) and lead to the activation of NADPH oxidase [4], [5]. Activated NADPH oxidase further catalyzes the transfer of electrons from NADPH to O_2_ [6]. While the role of NADPH oxidase in biological processes is well defined, the mechanisms that regulate the expression of the subunits of the NADPH oxidase and ROS homeostasis are still incompletely understood.

ROS play contradictory roles in cells. It is harmful or beneficial depending on its concentration and the cellular environment. In phagocytic cells, the NADPH oxidase complex is inactive under physiological conditions, while high levels of ROS production by the NADPH oxidase complex are essential for microbial killing [7]. Small amounts of ROS produced by nonphagocytic NADPH oxidase act as second messengers and influence redox-sensitive signal transduction pathway such as the mitogen-activated protein kinases (MAPKs) [8], [9]. However, when NADPH oxidase is upregulated, excess ROS may lead to oxidative damage, which is involved in tumor pathogenesis [10], [11], tumor growth, hypertension [12], and diabetic nephropathy [13]. In a biological sense, ROS are kept homeostasis through constantly production by many normal cellular events and counteraction by several antioxidant proteins [9].

The human protein HSCARG (also named NMRAL1, NmrA-like family domain containing protein 1) has been identified as a NADPH sensor. Our previous studies show that HSCARG forms an asymmetrical dimer with one subunit occupied by one NADP molecule and the other empty. In response to changes of intracellular NADPH/NADP^+^ levels, HSCARG shows conformational change and subcellular redistribution [14]. HSCARG is involved in the regulation of nitric oxide (NO) production through repression of the activity of argininosuccinate synthetase (AS) in response to changes of intracellular NADPH/NADP^+^ levels [14], [15]. Besides, HSCARG is essential for cell viability [15]. And more importantly, HSCARG is involved in the NF-κB signaling pathway through suppressing IKKβ phosphorylation [16]. Because HSCARG is an oxidative sensor, it is interesting to know if HSCARG regulates intracellular redox balance.

In this study, we investigated the effect of HSCARG on cellular ROS generation, and further elucidated the molecular mechanism by which HSCARG regulates ROS generation. We demonstrate that HSCARG downregulates ROS generation through regulating the expression of p47phox, a subunit of NADPH oxidase, and HSCARG decreases the expression of p47phox through inhibition of NF-κB activity.

## Results

### HSCARG Inhibits Cellular ROS Generation

**Figure 1:**
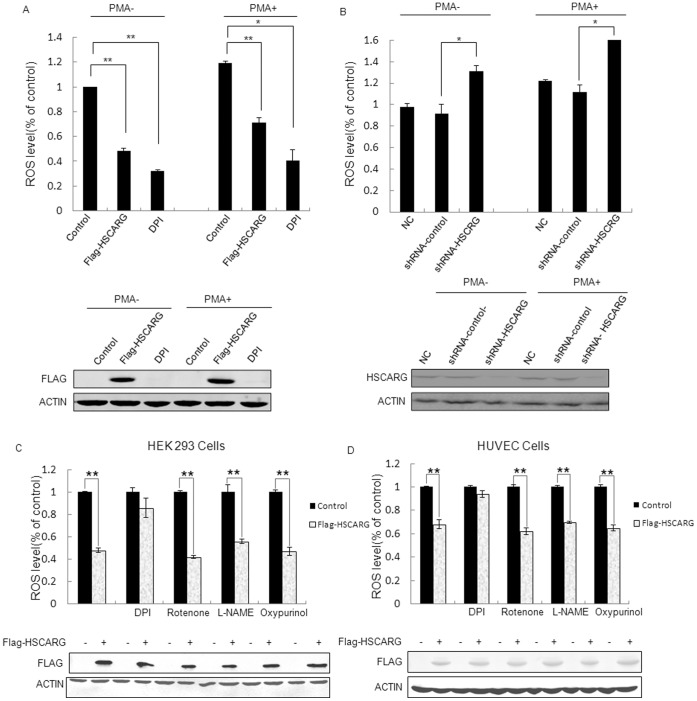
HSCARG regulates ROS generation in HEK293 cells. (**A**) HEK293 cells were transfected with 2 µg empty vector pRK-FLAG or 2 µg pRK-FLAG -HSCARG for 48 h, and then stimulated with 50 nM PMA for 30 minutes before harvested. Intracellular ROS production was measured by flow cytometry in cells labeled with DCF-DA. The expression of HSCARG was confirmed by western blot analysis using monoclonal anti-Flag antibody. (**B**) HEK293 cells were transfected with shRNA-HSCARG, pGCsi-nonsilence for 72 h, and then stimulated with 50 nM PMA for 30 minutes before harvested. And intracellular ROS production was measured by flow cytometry in cells labeled with DCF-DA experiments per group. Knockdown of HSCARG expression was examined by western blot analysis using polyclonal anti-HSCARG antibody. (**C, D**) HEK293 cells (**C**) and HUVECs (**D**) were transfected with 2 µg empty vector pRK-FLAG or 2 µg pRK-FLAG-HSCARG for 48 h, and then treated with specific inhibitors of ROS generating system including DPI (the inhibitor of NADPH oxidase, 5 µmol/l); L-NAME (the inhibitor of nitric oxide synthases, 100 µmol/l); rotenone (the inhibitor of mitochondrial respiratory chain, 2 µmol/l) and oxypurinol (the inhibitor of xanthine oxidase, 100 µmol/l) for 1 h. Intracellular ROS production was measured by flow cytometry in cells labeled with DCF-DA. Data are presented as mean ± S.E. of triplicate samples from a representative experiment (n = 3). Each experiment was repeated for at least three times. *p* value was ascertained by the Student’s *t* test (*p<0.05 vs. control, **p<0.01 vs. control).

To determine the effects of HSCARG on cellular ROS generation, human embryonic kidney 293 (HEK293) cells were transfected with pRK-Flag-HSCARG or empty plasmid pRK-Flag, and ROS generation was measured and compared. Cell treated with diphenyleneiodonium (DPI), a NOX family inhibitor, was used as a positive control. As expected, cells treated with DPI repressed ROS production significantly (Fig. 1A). Similarly, overexpression of HSCARG obviously decreased the production of ROS by about 40% when compared to that of control cells (Figure 1A). On the contrary, when HSCARG was knocked down in HEK293 cells, the production of ROS increased about 32% compared with control cells (Fig. 1B). Consistently, when cells were acutely exposed to 50 nM PMA, a PKC activator, for 30 min, similar results were observed in HEK293 cells transfected with plasmid of HSCARG or treated with DPI. ROS reduced significantly in HSCARG-transfected cells (Fig. 1A), while in HSCARG-depleted cells, ROS increased significantly (Fig. 1B).

To clarify that if HSCARG specifically inhibits NADPH oxidase-induced ROS, we examined the effect of HSCARG on ROS generation in HEK293 cells treated with the following different specific inhibitors of ROS-generating systems: diphenyleneiodonium chloride (DPI, the inhibitor of NADPH oxidase); NG-nitro-L-arginine methyl ester hydrochloride (L-NAME, the inhibitor of nitric oxide synthases); rotenone (the inhibitor of mitochondrial respiratory chain) and oxypurinol (the inhibitor of xanthine oxidase). Our results showed that compared with the control cells transfected with empty vector pRK-Flag, ectopic HSCARG expression still inhibited ROS generation significantly under treatment of L-NAME, rotenone and oxypurinol. While in cells treated with DPI, HSCARG lost its inhibitory function on ROS generation (Fig. 1C). Similar results were also observed in primary HUVEC cells (Fig. 1D). These results indicate that HSCARG mainly inhibits NADPH oxidase-induced ROS.

### HSCARG Inhibits Protein Expression of p47phox

**Figure 2:**
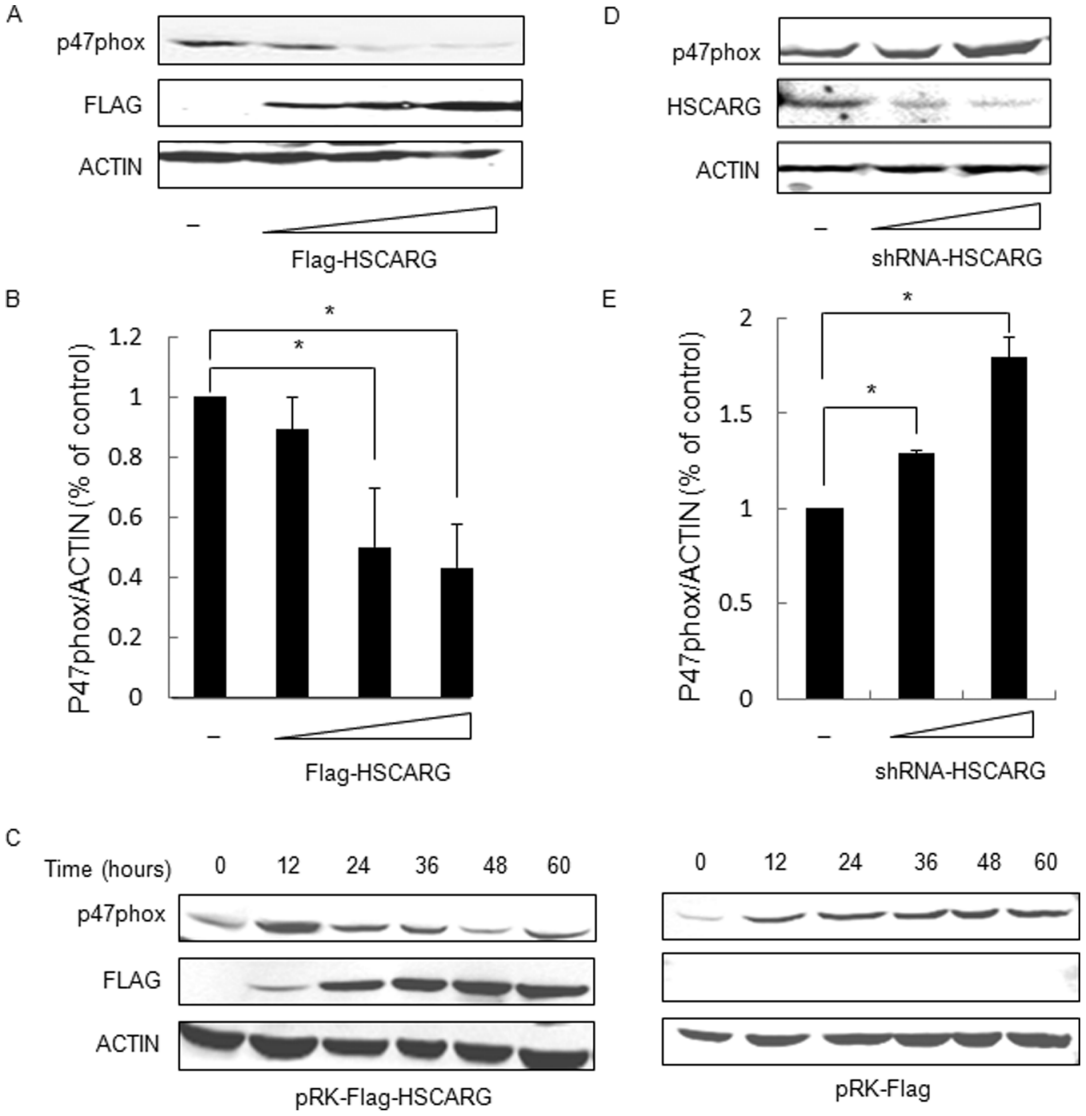
HSCARG inhibits the protein expression of p47phox in a dose/time dependent manner. (**A**) Various amounts of HSCARG (0, 0.5, 1.0, 2.0 µg) were transfected into HEK293 cells, and the change of endogenous p47phox protein was examined by western blot analysis using anti-p47phox antibody. (**B**) Band intensities of p47phox protein level were shown, which were calculated and compared to that of non-transfected cells. (**C**) The effect of HSCARG on p47phox protein in a time-dependent manner. HEK293 cells were transferred with 2.0 µg HSCARG (left) or control empty vector (right), and the protein levels of endogenous p47phox were examined at different time points (0, 12, 24, 36, 48, 60 h) by western blot analysis using anti-p47phox antibody. (**D**) Various amounts of shRNA-HSCARG (0, 1.0, 2.0 µg) were transfected into HEK293 cells, and the change of endogenous p47phox protein was examined by western blot analysis using anti-p47phox antibody. (**E**). Band intensities of p47phox protein level were shown, which were calculated and compared to that of non-transfected cells. Data are presented as mean ± S.E.,n = 3 independent experiments (*p<0.05 vs. control).

To investigate the molecular mechanism in which HSCARG regulates ROS generation, we examined whether HSCARG affected ROS production through interacting with the subunits of NADPH oxidase or regulating the expression of NADPH oxidase. We initially investigated whether HSCARG interacted with the cytosolic components of NADPH oxidase, p47phox and p67phox, by co-immunoprecipitation analysis (co-IP). No visible interaction could be detected (data not shown). Then we examined the effect of HSCARG on the expression of the components of NADPH oxidase. Obvious effect observed only on p47phox but not for p67phox, p22phox, p40phox. As shown in Figure 2A, the protein expression of p47phox was downregulated by HSCARG in a concentration dependent pattern. The band intensities were quantified with ImageQuant software. The result of quantitative analysis showed that overexpression of HSCARG in HEK293 cells decreased protein level of p47phox by about 45% (Fig. 2B). Besides, we examined the changes of p47phox protein levels at different time points after HSCARG transfection. The result showed that HSCARG decreased protein level of p47phox in a time-dependent manner (Fig. 2C). Moreover, in cells that HSCARG was knocked down, the protein expression of p47phox was increased (Fig. 2D). Quantitative analysis showed that the protein level of p47phox increased about 1.7 folds in HSCARG-depleted cells (Fig. 2E). These data indicate that HSCARG inhibits protein expression of p47phox.

### HSCARG Inhibits ROS Generation through Decreasing p47phox Expression

**Figure 3:**
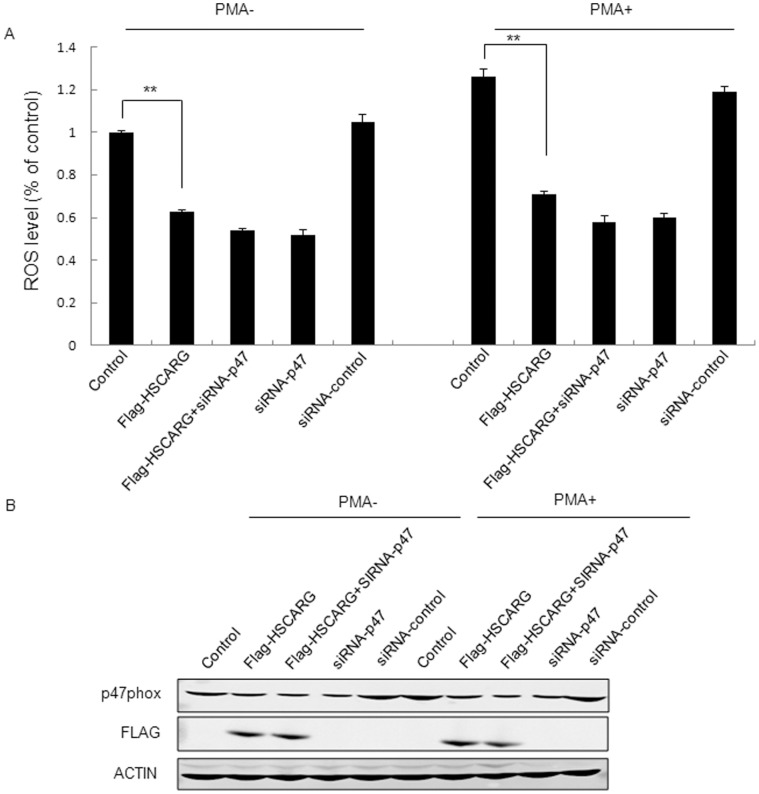
Regulation of ROS generation by HSCARG is dependent on p47phox. (**A**) HEK293 cells were transfected with siRNA-p47phox or siRNA–control for 24 hours, and then transfected with the indicated plasmids for another 48 hours. Before harvested, the cells were treated with 50 nM PMA for 30 min. After labeled with DCF-DA, cellular ROS generation was detected using flow cytometry. (**B**) The expression of the transfected plasmids was confirmed by western blot analysis using indicated antobodies. The results are mean ± S.E. of three different experiments (**p<0.01 vs. control).

To further explore whether HSCARG downregulated ROS generation through inhibition of p47phox expression, we investigated the effect of HSCARG on ROS production in cells with either depleted p47phox or wild-type p47phox. The results showed that, compared with control cells transfected with empty vector, overexpression of HSCARG obviously inhibited cellular ROS production (Fig. 1A, C, D and Fig. 3A). However, when p47phox was depleted by siRNA treatment, overexpression of HSCARG showed no obvious effect on ROS production (Fig. 3A, B). PMA treatment led to the similar result (Fig. 3A). These data indicate that HSCARG inhibits ROS production through decreasing the expression of p47phox.

### HSCARG Decreases mRNA Expression of p47phox

**Figure 4:**
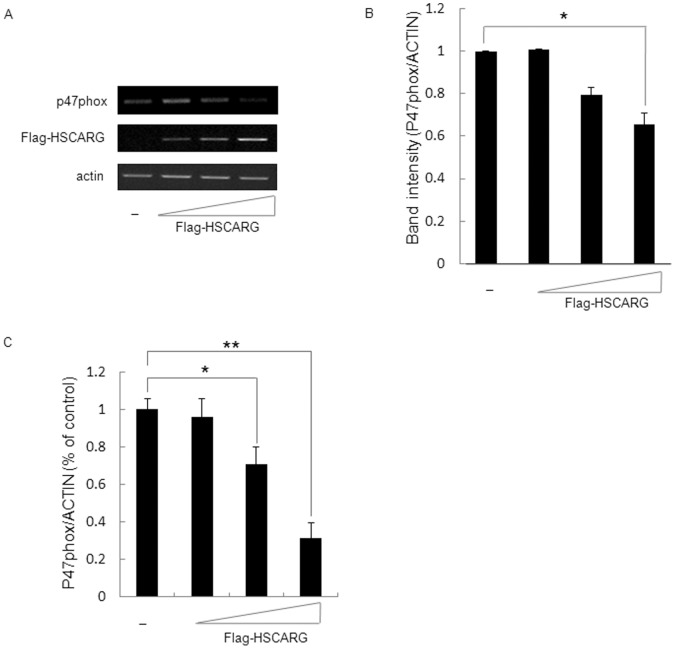
HSCARG inhibits mRNA of p47phox. (**A**) The effect of HSCARG on mRNA level of p47phox. Various amounts of pRK-Flag-HSCARG (0, 0.5, 1.0, 2.0 µg) were transfected into HEK293 cells, and the mRNA levels of p47phox were measured by RT-PCR. Actin was used as a loading control. (**B**) Band intensities of p47phox mRNA level were shown, which were calculated and compared to that of non-transfected cellls. (**C**) Various amounts of HSCARG (0, 0.5, 1.0, 2.0 µg) were transfected into HEK293 cells, and the mRNA levels of p47phox were measured by quantitative real-time PCR. Values are mean ± S.E. of 3 different experiments (*p<0.05 vs. control, **p<0.01 vs. control).

To investigate whether HSCARG also attenuate mRNA expression of p47phox, mRNA level of p47phox was accessed and compared in cells transfected with empty vector or different concentrations of HSCARG expression vector. The results showed that mRNA of p47phox was downreguleted by HSCARG in a dose dependent manner (Fig. 4A). Quantitative analysis of band intensity with ImageQuant software showed that the mRNA level of p47phox decreased by about 32% when HSCARG was overexpressed in HEK293 cells (Fig. 4B). Meanwhile, we screened the mRNA change of other subunits of NADPH oxidase (p22phox, p67phox, p40phox, rac1, nox2, and nox4) after overexpression of HSCARG. No obvious alteration detected for these subunits (Fig. S1). Quantitative real-time PCR was performed to further confirm the effect of HSCARG on p47phox mRNA expression. The results showed that p47phox mRNA was down-regulated by HSCARG in a does-depended manner. And it was decreased by about 65% in HEK293 cells with ectopic HSCARG expression (Fig. 4C). These data indicate that HSCARG inhibits p47phox mRNA expression.

### HSCARG Reduces p47phox Promoter Activity

**Figure 5:**
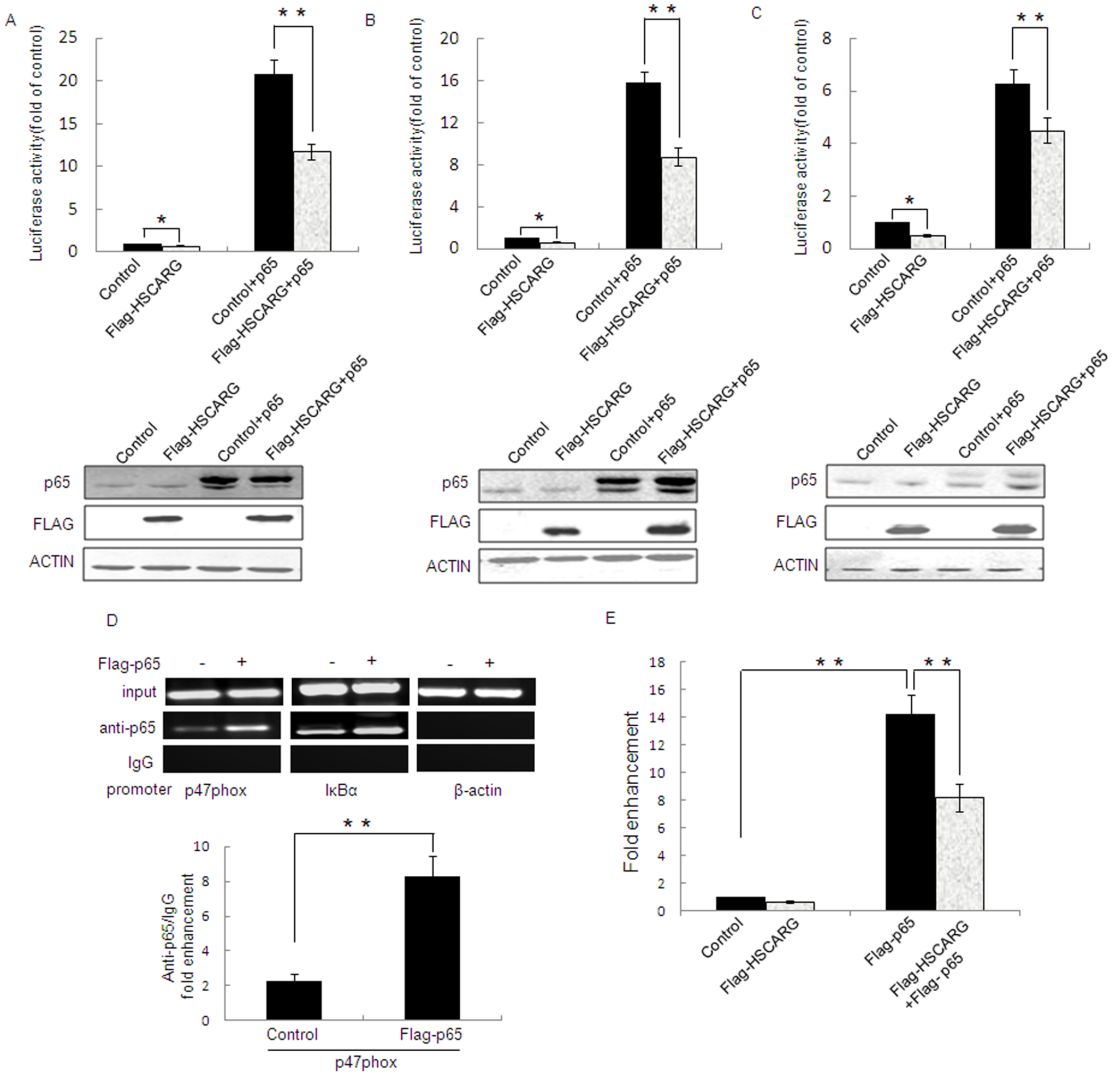
HSCARG reduces p47phox Promoter Activity. The activity of p47phox promoters -2151 (**A**), −224 (**B**), and −86 (**C**) was examined by luciferase report assay in HEK293 cells transfected with 2 µg HSCARG, pRK-Flag empty vector, pRK-Flag-p65 or pRK-Flag-p65 with pRK-Flag-HSCARG for 48 h, respectively. The expressions of HSCARG and p65 were confirmed by western blot analysis using monoclonal anti-Flag antibody and monoclonal anti-p65 antibody. (**D**) NF-κB can be recruited to the region from −224 to −1 bp in the p47phox promoter. HEK293 cells were transfected with Flag-p65, pRK-Flag was used as a control. ChIP assay was performed with polyclonal anti-p65 or rabbit anti-IgG (isotype control) antibodies. The immunoprecipitated p47phox promoter (left panel), IκBα promoter (middle panel), or β-actin promoter (right panel) were analyzed by PCR. Quantification of p47phox promoter from ChIP DNA was shown (**p<0.01 vs. control). (**E**) ChIP assay was quantified by real-time quantitative PCR. HEK293 cells were transfected with p65, HSCARG, and p65 with HSCARG expression plasmids, respectively, pRK-Flag empty vector was used as a control. Values are mean ± S.E. of 3 different experiments (*p<0.05 vs. control, **p<0.01 vs. control).

p47phox luciferase reporter constructs, −2151, −224, and −86, have been demonstrated to have strong p47phox promoter activity [17]. To clearly understand the regulatory mechanism of HSCARG on the expression of p47phox, we examined the effect of HSCARG on the activity of these p47phox promoter– luciferase reporter constructs by luciferase report assay. The results showed that overexpresison of HSCARG reduced promoter activity of p47phox −2151, −224, and −86 by about 25.12%,37.40%,50.28%, respectively (Fig. 5A, B and C). Because our previous study shows that HSCARG inhibits TNFα-induced NF-κB activity [16], and p47phox is one of the downstream substrates of NF-κB, we further detected the effect of NF-κB on p47phox promoter activity. As expected, the activities of the three p47phox promoter constructs increased 6.25,15.80 and 21.00 fold, respectively, in cells transfected with p65 (the main subunit of NF-κB) (Fig. 5A, B and C), and co-overexpression of HSCARG reduced p65-induced p47 activity by about 44.00%, 44.45%, 28.18% for −2151, −224, and −86 constructs, respectively (Fig. 5A, B and C).

The significant role of NF-κB in p47phox promoter activity prompted us to examine whether p65 protein, the main component of NF-κB, could be recruited to p47phox promoter. To this end, a chromatin immunoprecipitation (ChIP) assay was performed using anti-p65 antibody. As p65 is well established to bind to and regulate IκBα [18], p65 ChIP signal on the promoter of IκBα was served as a positive control. The result showed that precipitation of p65 gave the signal for the 200–300 bp region containing NF-κB binding site of IκBα (Fig. 5D, middle), similarly, precipitation of p65 gave the signal for the 250 bp region containing −224 to −1 bp of p47 promoter (Fig. 5D, left), and the signals were enhanced significantly in cells transfected with pRK-Flag-p65. Further quantification of p47phox promoter from ChIP DNA by real-time quantitative PCR showed that p65 ChIP signal was about 2.3-fold of that of control IgG ChIP, while it was 8.3-fold after p65 overexpression (Fig. 5D, lower). These results indicate that p65 is indeed present in the p47phox promoter *in vivo*. Overexpression of p65 resulted in an increase of p65 ChIP signal about 14-fold of that of control without p65 overexpression, and this p65-induced p47phox promoter activity decreased significantly in cells transfected with HSCARG (Fig. 5E). These results indicate that NF-κB could be recruited to the p47phox promoter, up-regulates p47phox activity and which could be decreased by HSCARG.

### HSCARG Decreases p47phox Expression through Inhibiting NF-κB Activity

**Figure 6:**
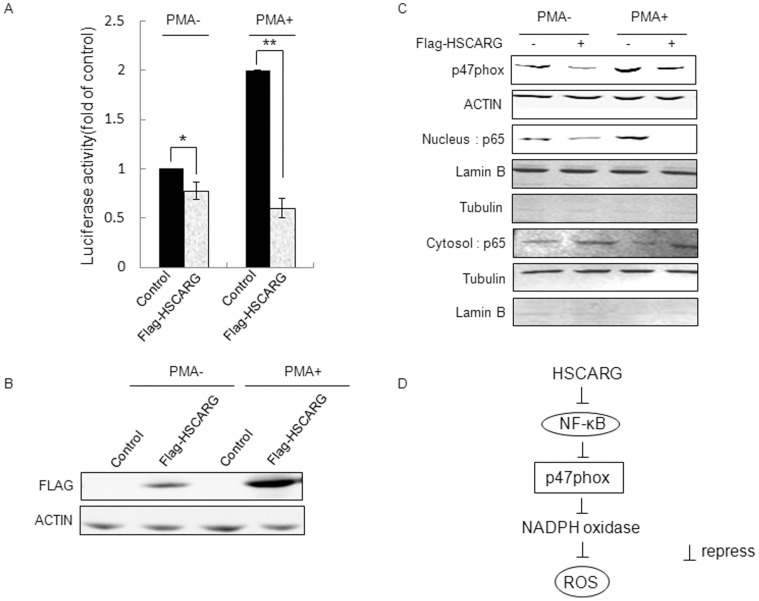
HSCARG inhibits p47phox expression through downregulating NF-κB activity. (**A**) NF-κB activity was examined by the luciferase report assay in HEK293 cells transfected with 2 µg pRK-Flag-HSCARG or pRK-Flag empty vector for 48 h, in the absence or presence of PMA treatment. (**B**) The expression of HSCARG was confirmed by western blot analysis using monoclonal anti-Flag antibody. (**C**) The effects of HSCARG on p65 nuclear translocation and p47phox expression in HEK293 cells treated with or without PMA. HEK293 cells were transfected with pRK-Flag-HSCARG or empty pRK-Flag vector, and then nuclear and cytoplasmic extracts were prepared using Nuclear and Cytoplasmic Extraction Reagents from Thermo Scientific. Extracts were then fractioned by SDS-PAGE and analyzed by immunoblotting using indicated antibodies. Lamin B and α-tubulin were used as nuclear and cytoplasmic loading controls, respectively. (**D**) The pathway through which HSCARG downregulating ROS production. HSCARG inhibits NF-κB activity, which downregulates the expression of p47phox, the subunit of NADPH oxidase. Attenuated p47phox activity further decreases ROS generation.

To further understand the regulation of HSCARG on p47phox, we investigated whether HSCARG decreased p47phox expression through inhibiting NF-κB activity. We confirmed the inhibitory effect of HSCARG on endogenous NF-κB activity by luciferase reporter assay (Fig. 6A, B). Meanwhile, we found that nuclear translocation of p65 was inhibited and the expression of p47phox was down-regulated in HSCARG–overexpressed cells (Fig. 6C). Similar results were observed in cells treated with PMA. As expected, PMA promoted p65 nuclear translocation, increased NF-κB activity, and stimulated the expression of p47phox (Fig. 6C). And overexpression of HSCARG repressed PMA-stimulated NF-κB activity by about 70% (Fig. 6A, B). Consistently, nuclear translocation of p65 was inhibited and the expression of p47phox was obviously down-regulated as well (Fig. 6C). These data together with the reporter assay shown in Figure 5 indicate that HSCARG inhibits the expression of p47phox through preventing nuclear translocation of p65 and decreasing NF-κB activity (Fig. 6D).

## Discussion

HSCARG protein is recently identified as an oxidative sensor, which down-regulates nitric oxide synthesis by reducing argininosuccinate synthetase activity in response to changes of intracellular redox states [15]. In this study, we elucidated the function of HSCARG in regulation of ROS homeostasis. We demonstrated that HSCARG inhibits ROS production through downregulating the expression of p47phox, a cytoplasmic subunit of NADPH oxidase.

We first found that HSCARG obviously decreased cellular ROS generation, and depletion of HSCARG increased ROS production. Because NADPH oxidase is the main source of ROS, we then detected whether HSCARG repressed ROS production through specifically regulating activity of NADPH oxidase. Treatment of cells with different inhibitors as shown in Figure 1C and D confirmed that HSCARG specifically inhibits NADPH oxidase-induced ROS. According to previous studies, the activity of NADPH oxidase is regulated through various processes, including adjustment of the PKC activity [19]; regulation of phosphorylation of NADPH oxidase components; and regulation of expression level of NADPH oxidase components [5], [7], [20]–[22]. We found that HSCARG down-regulated protein levels of p47phox in a concentration- and time- dependent manner. Knockdown of p47phox resulted in a lost of the effect of HSCARG on ROS generation, indicating that HSCARG inhibits NADPH oxidase activity through downregulating NADPH oxidase component p47phox protein expression.

Our previous studies show that HSCARG inhibits the activity of transcription factor NF-κB by suppressing IKKβ phosphorylation [16], [23]. As mRNA of p47 was down-regulated by HSCARG, we speculated that HSCARG decreased the expression of p47phox through inhibiting NF-κB activity. As a transcription factor, the p65:p50 heterodimer of NF-κB is thought to be responsible for the expression of many downstream genes including subunits of NADPH oxidase [21], [22], [24]. Pharmacological inhibitors of NF-κB activation block TNF-α induced up-regulation of p47phox and result in the reduction of corresponding proteins and decrease ROS generation [21], [22]. Here through luciferase reporter assay and ChIP assay, we investigated the regulation of p47phox promoter activity by HSCARG and p65. Our results suggested that p65 dramatically induced p47phox promoter activity, and which could be reduced by HSCARG. To ascertain the NF-κB binding site of p47phox promoter, electrophoretic mobility shift assay (EMSA) was performed by using −168 to −121 bp of p47phox promoter which was predicted to be potential binding sites in website www.gene-regulation.com as a probe, and −69 to −11 bp of p47phox promoter was used as a control. Competition experiment by preincubating 100 fold of non-labeled probe with nuclear extracts was also carried out. We observed the shifted complex, which were weakened when 100 fold non-labeled probes were used. Addition of polyclonal anti-p65 antibody also inhibited binding, but the supershift band to identify the specific transcriptional factor could not be detected (Fig. S2). It is worth to study whether p65 is recruited to the promoter of p47phox with the help of other protein(s) in the future.

It is also reported that PMA induces the nuclear transloaction of p65 and promotes NF-κB activation which further up-regulates the expression of downstream genes [25]. We therefore tested the effects of HSCARG on NF-κB activity, p65 nuclear translocation, and p47phox expression in cells treated with or without PMA. Our results confirmed the above speculation. Here we found that PMA treatment promoted more p65 translocating into nucleus and increased NF-κB activity, which is consistent with previous report. And PMA also stimulated p47phox expression as expected. In cells treated with or without PMA, we all found that HSCARG inhibits NF-κB activity by preventing p65 nuclear translocation, which subsequently decreases the expression of p47phox. This finding suggests that HSCARG inhibits p47phox expression through down-regulating NF-κB activity.

It has been reported that NADPH oxidase-induced ROS involved in disease pathogenesis including inflammation [26], host defense [27], diabetic nephropathy [28] and so on. Therefore, it is worthy to elucidate whether HSCARG involved in such diseases through regulating ROS in the future.

In conclusion, here, we demonstrate that HSCARG is involved in regulation of ROS homeostasis by inhibiting NADPH oxidase activity. Downregulation of NADPH oxidase activity by HSCARG is through inhibiting NF-κB activity, which further decreases the mRNA and protein expressions of p47phox, and thus inhibits ROS generation. Our study identifies HSCARG as a negative regulator of ROS generation, and elucidates a possible mechanism by which HSCARG regulates NADPH oxidase activity and counterbalances ROS generation.

## Materials and Methods

### Cell Culture

Human embryonic kidney(HEK293)cell lines were cultured in Iscove’s modified Dulbecco’s medium (IMDM, Invitrogen, CA, USA) supplemented with 10% fetal bovine serum (Hyclone, Logan, UT, USA) at 37°C in a 5% CO_2_ incubator. Human Umbilical Vein Endothelial Cells (HUVECs, a gift from Dr. Jincai Luo) were cultured in M199 medium (Gibco,Grand Island, NY, USA) supplemented with 1% thymidine (Sigma-Aldrich, St. Louis, Missouri, USA), 20% FBS (Hyclone),10 mM HEPES, 5 ng/ml hFGF (Peprotech, Rocky Hill, New Jersey, USA), 2 mM L-Glutanmine (Sigma-Aldrich), at 37°C in a 5% CO_2_ incubator.

### Transfection

HEK293 cells (2×10^5^ cells/ml) were seeded on 6-well dishes, and transiently transfected with the indicated plasmids using MegaTran 1.0 transfection reagent (Origene, Rockville, MD, USA) according to manufacture’s protocol [29]. Cells were harvested at 48 hours after transfection with pRK-FLAG-HSCARG or pRK-FLAG empty vectors. To knock down the expression of p47phox or HSCARG, HEK293 cells (2×10^5^ cells/ml) were seeded on 6-well dishes, and transfected with siRNA targeting p47phox mRNA (Santa Cruze, CA, USA) or shRNA-HSCARG (GeneChem, Shanghai, China) after 24 h after incubation, siRNA-control (Santa Cruze) or shRNA-control (GeneChem, Shanghai, China) were used as a control. And cells were collected at 72 hours after transfection. HUVECs (2×10^5^ cells/ml) were seeded on 6-well dishes and transiently transfected with the indicated plasmids.

### Western Blot Analysis

After specific treatments and cultured for indicated time, cells were lyzed in buffer containing 50 mM Tris-HCl (pH 7.5), 150 mM NaCl, 1% NP-40, 0.5% deoxidizing bile acid sodium, 0.1% Sodium dodecylsulfate (SDS) 100 mmol/L phenylmethylsulfonyl fluoride, and a commercial protease inhibitor cocktail (Calbiochem, San Diego, CA) for 40 minutes on ice. Cell lysates (40∼80 µg protein) were separated by 12% or 15% SDS–PAGE, transferred to derivatized polyvinylidene difluoride (PVDF) membrane (GE Health Care, Waukesha, WI, USA), and blocked with 5% non-fat dry milk. The membrane was then probed with antibodies at 4°C overnight or at room temperature for 2 hours, followed by incubating with IRDye 800CW secondary antibody (LI-COR Biosciences, NE, USA) for 50 min. The membrane was washed with Tris buffered saline with Tween-20 (TBST, pH8.0) three times and then scanned with ODYSSEY infrared imaging system.

### Determination of Intracellular Reactive Oxygen Species (ROS)

HEK293 cells and HUVECs were transfected with pRK-Flag-HSCARG for 48 hours with pRK- Flag as a control; or transfected with shRNA-HSCARG for 72 hours with shRNA-control as a control. Before harvest, cells were incubated with 10 µmol/L of fluoroprobe 20,70-dichlorodihydro- fluorescein diacetate (DCFH-DA) (Beyotime, Jiangsu, China)for 30 min at 37°C (This compound is rapidly taken up by the cells and converted by intracellular esterase to 20, 70-dichlorodihydrofluorescein (DCF), which can be visualized by fluorescence at 525 nm) [30]. The labeled cells transfected with pRK-Flag and pRK-Flag-HSCARG were treated with different specific inhibitors (Sigma-Aldrich) of ROS-generation system including DPI (NOX NADPH oxidase, 5 µmol/l), L-NAME (nitric oxide synthases, 100 µmol/l), rotenone (mitochondrial respiratory chain, 2 µmol/l) and oxypurinol (xanthine oxidase, 100 µmol/l) for 1 hour and then the DCF fluorescence was measured by flow cytometry.

### RNA Extraction

HEK293 cells were transiently transfected with the indicated plasmids. At 48 hours after transfection, the total RNA was extracted using Trizol (Invitrogen, Carlsbad, CA) according to manufacture’s instruction [31]. The extracted RNA was resolved in RNase-free water, and stored at −80°C for further uses.

### Reverse Transcription (RT) PCR

RT-PCR was performed using a RT-PCR Kit (Promega, Madison,USA) according to manufacturer’s instruction [32]Primers used in RT-PCR are as follows:

nox2, 5′-GAATTGTACGTGGGCAGACCG (forward primer), 5′-GGACACCTTTG GGCACTTGAC (reverse primer); nox4, 5′-TTAGATACCCACCCTCCCGGCT (forward primer), 5′-AAATGATGGTGACTGGCTTAT (reverse primer); p22phox, 5′-GATGGTGGCCAGCAGGAAG (forward primer), 5′-CAGTGGGCCATGTGGG CCAACG (reverse primer); p40phox, 5′-CGGATACCTGCCCTCAACGCCTAC (forward primer), 5′-CATCCGACAGCAGCCGAACCA (reverse primer); p47phox, 5′-GCTCCCCACGGACAACCAGAC (forward primer), 5′-TCTTCTCCACGACC TCCACCAC (reverse primer); p67phox, 5′-ACGAGGGATGCTCTACTACCA (forward primer), 5′-CCTCTGGTTGGGTAGCCTCAT (reverse primer); rac1, 5′-TG AATCTGGGCTTATGGGATAC (forward primer), 5′-CCGAGCACTCCAGGTATTT TAC (reverse primer). Temperature gradient PCR instrument was used to optimize the annealing temperatures for the subunits of NADPH oxidase.

### Quantitative Real-time PCR

Quantitative real-time PCR was performed using a Real-time qPCR Kit (BIO-RAD, Hercules, CA, USA) according to manufacturer’s instruction. Primers used in real-time PCR are the same as the RT-PCR.

### Luciferase Reporter Assay

HEK293 cells (2×10^5^ cells/ml) were seeded on 24-well dishes and transiently transfected with 200 ng of NF-κB luciferase reporter plasmid or p47phox promoter reporter constructs (pGL3-p47-2151 construct, pGL3-p47-224 construct, pGL3-p47-86 construct, [17]) and other plasmids as indicated. 20 ng of *Renilla* luciferase reporter plasmid was cotransfected into each well as an internal control. Empty control vector was added to equalize the amount of total DNA. At 24 hours after transfection, the cells were harvested and luciferase assay was assessed using a dual-luciferase reporter assay system (Promega, Madison,USA) [33]. All reporter assays were performed in triplicate for at least three independent experiments and data from one representative experiment were shown here. The means ± S.E. are shown in the figures.

### Chromatin Immunoprecipitation (ChIP)

5×10^6^ HEK293 cells were crosslinked with 1% formaldehyde (Sigma-Aldrich, St. Louis, Missouri, USA) for 8 min. The reaction was terminated by adding 0.125 M glycine. Cell lysates were prepared and chromosomal DNA was sonicated to reach average sizes around 200–500 bps. The lysate was immunoprecipitated with either rabbit IgG or an anti-p65 antibody (Bioworld, Atlanta, Georgia, USA). Upon reversal of the crosslinking, the purified DNA fragments were PCR-amplified for p47phox promoter, with IκBα as a positive control and actin as a negative control, then real time quantitative PCR was performed to analyze the p47phox promoter. ChIP -DNA was used for PCR with the primers as follows: human p47 promoter (−224 to −1): 5′-AAGTGTAAACCCTTTTCCTT(forward primer), 5′GACTGGGTGGCCTCCAG TG (reverse primer); human IκBα promoter (−316 to −15) [18]: 5′-GACGACCCCA ATTCAAATCG(forward primer), 5′–TCAGGCTCGGGGAATTTCC(reverse primer); and human β-actin promoter (−980 to −915) [18]: 5′ –TGCACTGTGCGGCGAAGC (forward primer), 5′-TCGAGCCATAAAAGGCAA (reverse primer).

### Electrophoretic Mobility Shift Assay

5×10^6^ HEK293 cells transfected with pRK-Flag-p65 or pRK-Flag were collected and incubated with buffer A (10 mM HEPES (pH 7.9), 50 mM NaCl, 1.5 mM MgCl_2_, 1 mM dithiothreitol (DTT) and 0.5 mM phenylmethylsulfonyl fluoride (PMSF)) for 30 min on ice, and then centrifuged at 14,000 rpm, 4°C for 10 min. The crude nuclear pellet was resuspended in 0.2 ml of buffer B (20 mM HEPES, pH 7.9, 1.5 mM MgCl_2_, 420 mM NaCl, 1 mM DTT, 1 mM EDTA, 1 mM PMSF, and 25% glycerol) and incubated for 30 min on ice. The suspension was centrifuged at 14,000 rpm and 4°C for 15 min. The supernatant (nuclear protein) was collected and stored at −80°C. Oligonucleotide probes were labeled with γ-^32^P ATP (PerkinElmer,California,USA) and T4 polynucleotide kinase (Takara, Dalian, China), which were then annealed in annealing buffer by heating at 95°C for 5 min and cooling at room temperature. For gel shift assays, 6 µg nuclear extract was incubated with the labeled DNA probe (30,000 cpm) in binding buffer containing 10 mM HEPES (pH 7.9), 5 mM MgCl_2_, 1 mM DTT, 1 mM EDTA, 50 mM NaCl, 5% glycerol, 1 mg/ml bovine serum albumin, and 2 mg of salmon sperm DNA (Sigma-Aldrich, St. Louis, Missouri, USA) for 15 min at room temperature. For supershift assays, 2 µg of polyclonal anti-p65 antibody (Bioworld, Atlanta, Georgia, USA) was added to the reaction solution and continuously incubated for 15 min at room temperature. Competition assays were carried out by preincubating the above reaction mixture with competitor DNA for 15 min at room temperature before addition of the labeled probe. Samples were loaded on 5% non-denaturing polyacrylamide gels. The DNA sequences used as probes in the promoter of p47phox are as follow. Probe 1, 5′-GTTTACACCCTGCAAGCCT GAAGAGTCCCCAGAAACTGAAAGAAGAAGC; Probe 1 mutation construct, 5′-GTTTACACCCTGCAAGCATTAATACTTACCCAGAAACTGAAAGAAGAA GC.

### Statistical Analysis

Each experiment was performed at least three times, and all values were represented as mean ± S.E. The Student’s *t* test was used to determine significance, requiring *p*<0.05 for statistical significance.

## Supporting Information

Figure S1 **HSCARG has no effect on mRNA level of the other subunits of NADPH oxidase.** Various amounts of pRK-Flag-HSCARG (0, 0.5, 1.0, 2.0 µg) were transfected into HEK293 cells, and the mRNA levels of nox2, nox4, p22phox, p40phox, p67phox, rac1 were measured by RT-PCR.
(TIF)

Figure S2 **The interaction between NF-κB and p47 promoter could not be detected by electrophoretic mobility shift assay (EMSA).** HEK293 cells were transfected with (+) or without (−) pRK-Flag-p65 for 48 h, and then the nuclear extracts were analyzed by EMSA. (**A**) −168 to−121 bp of p47phox promoter which predicted to contain potential NF-κB binding site was labeled withγ-^32^P-ATP. Supershift analysis was conducted with anti-p65. Lanes 1 to 7 indicate free probe, nuclear extracts, nuclear extracts+probe, nuclear extracts+probe, molar excess (1∶100), anti-p65 antibody+nuclear extracts+probe, mutation (1∶100), respectively. (**B**) −69 to −11 bp of p47phox promoter were used as a negative control. Lanes 1 to 6 indicate free probe, nuclear extracts, nuclear extracts+probe, nuclear extracts+probe, molar excess (1∶100), anti-p65 antibody+nuclear extracts+probe, respectively.
(TIF)
