# Supplementary figures and images for: HSCARG Inhibits NADPH Oxidase Activity through Regulation of the Expression of p47phox
Source: PLoS One. 2013 Mar 19;8(3):e59301. doi: 10.1371/journal.pone.0059301 (PMC3602244; doi:10.1371/journal.pone.0059301)

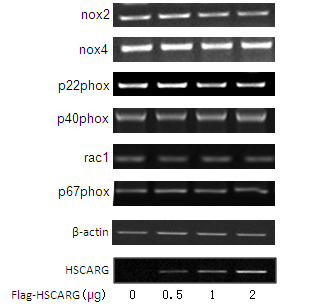

Supplement: Figure S1 — HSCARG has no effect on mRNA level of the other subunits of NADPH oxidase. Various amounts of pRK-Flag-HSCARG (0, 0.5, 1.0, 2.0 µg) were transfected into HEK293 cells, and the mRNA levels of nox2, nox4, p22phox, p40phox, p67phox, rac1 were measured by RT-PCR. (TIF) [file pone.0059301.s001.tif]

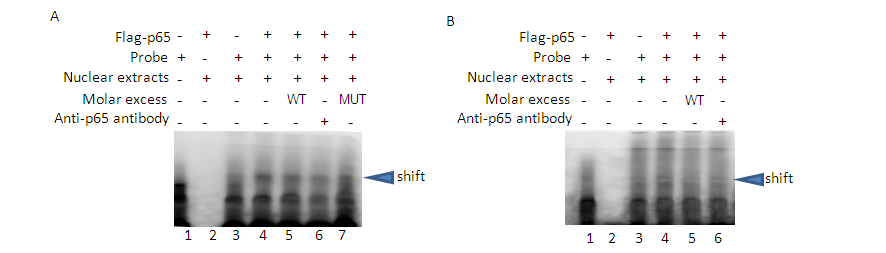

Supplement: Figure S2 — The interaction between NF-κB and p47 promoter could not be detected by electrophoretic mobility shift assay (EMSA). HEK293 cells were transfected with (+) or without (−) pRK-Flag-p65 for 48 h, and then the nuclear extracts were analyzed by EMSA. (A) −168 to−121 bp of p47phox promoter which predicted to contain potential NF-κB binding site was labeled withγ-32P-ATP. Supershift analysis was conducted with anti-p65. Lanes 1 to 7 indicate free probe, nuclear extracts, nuclear extracts+probe, nuclear extracts+probe, molar excess (1∶100), anti-p65 antibody+nuclear extracts+probe, mutation (1∶100), respectively. (B) −69 to −11 bp of p47phox promoter were used as a negative control. Lanes 1 to 6 indicate free probe, nuclear extracts, nuclear extracts+probe, nuclear extracts+probe, molar excess (1∶100), anti-p65 antibody+nuclear extracts+probe, respectively. (TIF) [file pone.0059301.s002.tif]
